# Rotavirus Genotypes in Hospitalized Children With Acute Gastroenteritis Before and After Rotavirus Vaccine Introduction in Blantyre, Malawi, 1997–2019

**DOI:** 10.1093/infdis/jiaa616

**Published:** 2020-10-09

**Authors:** Chimwemwe Mhango, Jonathan J Mandolo, End Chinyama, Richard Wachepa, Oscar Kanjerwa, Chikondi Malamba-Banda, Prisca B Matambo, Kayla G Barnes, Chrispin Chaguza, Isaac T Shawa, Martin M Nyaga, Daniel Hungerford, Umesh D Parashar, Virginia E Pitzer, Arox W Kamng’ona, Miren Iturriza-Gomara, Nigel A Cunliffe, Khuzwayo C Jere

**Affiliations:** Malawi-Liverpool-Wellcome Trust Clinical Research Programme, Blantyre, Malawi; Department of Medical Laboratory Sciences, College of Medicine, University of Malawi, Blantyre, Malawi; Department of Biomedical Sciences, College of Medicine, University of Malawi, Blantyre, Malawi; Malawi-Liverpool-Wellcome Trust Clinical Research Programme, Blantyre, Malawi; Department of Medical Laboratory Sciences, College of Medicine, University of Malawi, Blantyre, Malawi; Department of Biomedical Sciences, College of Medicine, University of Malawi, Blantyre, Malawi; Malawi-Liverpool-Wellcome Trust Clinical Research Programme, Blantyre, Malawi; Malawi-Liverpool-Wellcome Trust Clinical Research Programme, Blantyre, Malawi; Malawi-Liverpool-Wellcome Trust Clinical Research Programme, Blantyre, Malawi; Malawi-Liverpool-Wellcome Trust Clinical Research Programme, Blantyre, Malawi; Department of Medical Laboratory Sciences, College of Medicine, University of Malawi, Blantyre, Malawi; Centre for Global Vaccine Research, Institute of Infection, Veterinary and Ecological Sciences, University of Liverpool, Liverpool, United Kingdom; Malawi-Liverpool-Wellcome Trust Clinical Research Programme, Blantyre, Malawi; Department of Medical Laboratory Sciences, College of Medicine, University of Malawi, Blantyre, Malawi; Centre for Global Vaccine Research, Institute of Infection, Veterinary and Ecological Sciences, University of Liverpool, Liverpool, United Kingdom; Department of Immunology and Infectious Diseases, T. H. Chan School of Public Health, Harvard University, Boston, Massachusetts, USA; Genomics of Pneumonia and Meningitis, Wellcome Sanger Institute, Wellcome Genome Campus, Hinxton, United Kingdom; Malawi-Liverpool-Wellcome Trust Clinical Research Programme, Blantyre, Malawi; Department of Medical Laboratory Sciences, College of Medicine, University of Malawi, Blantyre, Malawi; Next Generation Sequencing Unit and Division of Virology, Faculty of Health Sciences, University of the Free State, Bloemfontein, South Africa; Centre for Global Vaccine Research, Institute of Infection, Veterinary and Ecological Sciences, University of Liverpool, Liverpool, United Kingdom; National Institute for Health Research Health Protection Research Unit in Gastrointestinal Infections, University of Liverpool, United Kingdom; Epidemiology Branch, Division of Viral Diseases, National Center for Immunization and Respiratory Diseases, Centers for Disease Control and Prevention, Atlanta, Georgia, USA; Department of Epidemiology of Microbial Diseases, Yale School of Public Health, Yale University, New Haven, Connecticut, USA; Malawi-Liverpool-Wellcome Trust Clinical Research Programme, Blantyre, Malawi; Department of Biomedical Sciences, College of Medicine, University of Malawi, Blantyre, Malawi; Centre for Global Vaccine Research, Institute of Infection, Veterinary and Ecological Sciences, University of Liverpool, Liverpool, United Kingdom; National Institute for Health Research Health Protection Research Unit in Gastrointestinal Infections, University of Liverpool, United Kingdom; Centre for Global Vaccine Research, Institute of Infection, Veterinary and Ecological Sciences, University of Liverpool, Liverpool, United Kingdom; National Institute for Health Research Health Protection Research Unit in Gastrointestinal Infections, University of Liverpool, United Kingdom; Malawi-Liverpool-Wellcome Trust Clinical Research Programme, Blantyre, Malawi; Department of Medical Laboratory Sciences, College of Medicine, University of Malawi, Blantyre, Malawi; Centre for Global Vaccine Research, Institute of Infection, Veterinary and Ecological Sciences, University of Liverpool, Liverpool, United Kingdom; National Institute for Health Research Health Protection Research Unit in Gastrointestinal Infections, University of Liverpool, United Kingdom

**Keywords:** rotavirus, genotypes, gastroenteritis, Malawi, surveillance, Africa

## Abstract

**Background:**

Rotavirus vaccine (Rotarix [RV1]) has reduced diarrhea-associated hospitalizations and deaths in Malawi. We examined the trends in circulating rotavirus genotypes in Malawi over a 22-year period to assess the impact of RV1 introduction on strain distribution.

**Methods:**

Data on rotavirus-positive stool specimens among children aged <5 years hospitalized with diarrhea in Blantyre, Malawi before (July 1997–October 2012, n = 1765) and after (November 2012–October 2019, n = 934) RV1 introduction were analyzed. Rotavirus G and P genotypes were assigned using reverse-transcription polymerase chain reaction.

**Results:**

A rich rotavirus strain diversity circulated throughout the 22-year period; Shannon (H′) and Simpson diversity (D′) indices did not differ between the pre- and postvaccine periods (H′ *P* < .149; D′ *P* < .287). Overall, G1 (n = 268/924 [28.7%]), G2 (n = 308/924 [33.0%]), G3 (n = 72/924 [7.7%]), and G12 (n = 109/924 [11.8%]) were the most prevalent genotypes identified following RV1 introduction. The prevalence of G1P[8] and G2P[4] genotypes declined each successive year following RV1 introduction, and were not detected after 2018. Genotype G3 reemerged and became the predominant genotype from 2017 onward. No evidence of genotype selection was observed 7 years post–RV1 introduction.

**Conclusions:**

Rotavirus strain diversity and genotype variation in Malawi are likely driven by natural mechanisms rather than vaccine pressure.

## Teaser

Combined G and P genotype diversity did not significantly change following rotavirus vaccine introduction in Malawi, but P genotype diversity increased in children aged >12 months. G3 rotaviruses reemerged and replaced G1P[8] and G2P[4] as the predominant genotype from 2017 onward.

Rotavirus remains the leading cause of severe, dehydrating diarrhea among young children globally [1]. Vaccination is the primary public health approach to reduce the burden of rotavirus disease, and >100 countries have introduced rotavirus vaccine into their immunization schedules (https://vaccineresources.org/rotavirus.php). There has been a notable reduction in global rotavirus diarrhea-associated mortality following vaccine introduction, from more than half a million deaths each year to an estimated 128 500 annual deaths among children <5 years of age. However, 258 million annual diarrhea episodes are still associated with rotavirus infection [2]. More than 90% of these cases occur in low- and middle-income countries (LMICs), where rotavirus vaccine effectiveness is lower (39%–66%) compared to high-income settings (>85%) [3, 4].

Since the introduction of the monovalent Rotarix G1P[8] rotavirus vaccine (RV1) into Malawi’s national immunization program on 29 October 2012 [4], there has been a 54.2% reduction in population rotavirus hospitalization incidence in children aged <5 years and a 33% reduction in infant mortality from all-cause diarrhea [5, 6]. Despite the positive impact of rotavirus vaccination in Malawi and other African countries, the burden of rotavirus disease remains high, and an estimated 20–49.9 deaths in every 100 000 children aged <5 years are still associated with rotavirus infection in the sub-Saharan region [2]. Given the greater rotavirus strain diversity in Africa [7, 8], there is a need to better understand the extent of heterotypic protection afforded by vaccination and the impact of vaccination on the diversity of circulating rotavirus strains.

Rotaviruses comprise their own genus within the Reoviridae family [9]. Their genome is composed of 11 segments of double-stranded RNA (dsRNA) enclosed in triple capsulated protein layers. With the exception of segment 11 that encodes 2 proteins in some species, the remainder of the rotavirus genome segments encode a specific viral protein; hence, there are 6 structural (VP1–VP4, VP6, and VP7) and 6 nonstructural (NSP1–NSP6) proteins [9]. Rotaviruses are classified into 9 groups (A – I) based on the properties of their VP6 protein. Group A rotaviruses are associated with >90% of human infections and can be further differentiated using a dual classification system that assigns G and P genotypes based on nucleotide sequence similarities of VP7 and VP4 encoding genome segments, respectively [9]. So far, 36 G and 51 P rotavirus genotypes have been identified (http://rega.kuleuven.be/cev/viralmetagenomics/virus-classification), of which G1P[8], G2P[4], G3P[8], G4P[8], G9P[8], and G12P[8] are the major global circulating strains in humans [10]. Unlike in high-income countries, a wide rotavirus strain diversity is present in African countries where atypical rotaviruses including genotypes G1P[4], G1P[6], G8P[4], G8P[8], and G6P[6] are also frequently characterized [11–17]. Although a consistent change in the pattern of strains circulating after the introduction of rotavirus vaccine has not yet been identified, a reduction in circulation of G1 rotaviruses and an increase in the detection of heterotypic strains [10], for instance G2P[4] rotaviruses in countries using RV1, has been reported in various settings [7, 18–20]. However, long-term studies are lacking from LMICs.

We have conducted rotavirus strain surveillance at the Queen Elizabeth Central Hospital (QECH) in Blantyre, Malawi, since 1997; genotypes G1P[8], G2P[4], G3[P4], G3P[8], G4P[8], G8P[6], G8P[8], and G12P[8] were the most frequently detected rotaviruses before and immediately following vaccine introduction [8, 11, 21–23]. To assess the impact of vaccine introduction on the diversity of circulating strains, we examined the trends in circulating rotavirus genotypes over a 22-year period (1997–2019), and compared the genotype diversities between rotaviruses that circulated before (1997–2012) and after (2012–2019) RV1 introduction.

## MATERIALS AND METHODS

### Rotavirus Surveillance Platform

We enrolled children <5 years old presenting to the QECH, Blantyre, with acute gastroenteritis (at least 3 looser-than-normal stools in a 24-hour period). QECH is the main referral hospital in the southern region of Malawi that serves 23 government primary health centers within Blantyre city, which has a population of >1.25 million with a median age of 17.4 years according to the Malawi 2018 population census (http://www.nsomalawi.mw).

We examined rotavirus genotypes identified from 2 periods of observation, reflecting pre- and postvaccine periods. Therefore, pre-RV1 genotypes were detected from July 1997 to October 2012, whereas post-RV1 genotypes were detected from November 2012 to October 2019. Enrollment criteria were similar in both periods, incorporating both inpatients and outpatients, and included documentation of vaccine status from handheld health passports in the postvaccine period as described previously [5, 11, 12, 21, 23–25].

### Rotavirus Detection and Genotyping

A minimum 20% stool suspension prepared in Rotaclone kit diluent buffer was used to screen for the presence of group A rotaviruses using a commercially available enzyme immunoassay (Rotaclone, Meridian Bioscience, Cincinnati, Ohio). Raw stool samples were stored at –80°C and were subsequently used to prepare 20% stool suspensions in phosphate-buffered saline for molecular assays. Rotavirus dsRNA was extracted from freshly prepared rotavirus-positive stool suspensions collected from 2011 to 2019 using the Viral RNA Mini-Kit (Qiagen, Hilden, Germany). Rotavirus dsRNA was reverse transcribed to complementary DNA (cDNA) using random primers (Invitrogen, Paisley, UK) and reverse transcriptase enzyme (Superscript III MMLV-RT, Invitrogen) [26]. The cDNA was used to assign G genotype (G1, G2, G3, G4, G8, G9, G10, G11, G12) and P genotype (P[4], P[6], P[8], P[9], P[10], P[11], P[14]) to rotavirus-positive samples using a multiplex heminested reverse-transcription polymerase chain reaction (RT-PCR) assay as previously described [27]. For samples collected between 1997 and 2009, a guanidine and silica gel method was used to extract rotavirus dsRNA from stool, and the RT-PCR did not include G11, P[9], or P[14] genotype–specific primers [11]. Nucleotide sequencing was undertaken on at least 10% of the characterized genotypes to confirm the RT-PCR–derived VP4 and VP7 genotypes on randomly selected, representative strains using established methods [21, 28], followed by online genotyping using VIPR (https://www.viprbrc.org).

### Rotavirus Genotype Diversity

Rotavirus genotype diversity during pre- and postvaccine periods was compared using the Simpson diversity index (D′) and Shannon diversity index (H′) [29]. In this context, Simpson diversity measures the probability that 2 rotavirus strains randomly selected from a sample will belong to the same G and P genotype combination, whereas the Shannon index quantifies the uncertainty in predicting the rotavirus genotype of an individual sample randomly selected from the dataset. We employed the Simpson diversity index as a Simpson reciprocal index (1 / D) [29]. Differentially abundant rotavirus genotypes were determined using IndVal, labdsv version 2.0_1, an indicator species analysis package in R [30].

### Statistical Analyses

Differences in genotype diversities between the pre- and postvaccine periods were calculated by Student *t* test or Wilcoxon signed-rank test. The χ ^2^ test or Fisher exact test were used to measure associations. Indicator species analysis was conducted to identify the genotypes that were significantly associated with a given group [31]. Statistical significance of observed indicator species values was established by permutation tests, and *P* values were corrected for multiple comparisons with the Benjamini–Hochberg procedure at 10% false discovery rate.

To further investigate relative statistical changes in circulating genotypes pre– and post–vaccine introduction in Malawi, we used multinomial logistic regression models, with genotype as the outcome and G1P[8] as the reference genotype. Model fitting included the following covariates: surveillance year (November–October), age in months, and sex. Multinomial odds ratios (MORs), 95% confidence intervals (CIs), and associated *P* values were calculated from the Wald test. Due to limited study recruitment between November 2009 and October 2011, these years were removed from the MOR analyses. All statistical analyses were performed in GraphPad Prism version 8 and R package version 3.3.2 software [30].

### Ethical Approval

Ethical approval was obtained from the National Health Sciences Research Committee, Lilongwe, Malawi (number 867) and the Research Ethics Committee of the University of Liverpool, United Kingdom (000490).

## RESULTS

### Rotavirus Genotype Diversity (July 1997–October 2019)

**Figure 1.**
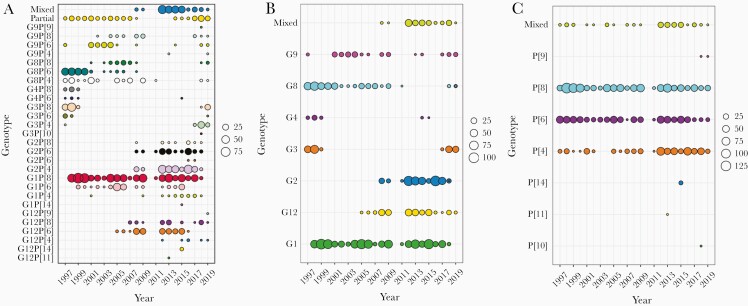
Trends of rotavirus strains from 1997 to 2019. *A*, Combined G and P genotypes of the genotyped samples. *B*, G genotypes only. *C*, P genotypes only. The size of the circle corresponds to the number of strains of that genotype detected in a given year.

 The percentage of stool samples in which rotaviruses were detected did not differ significantly before (33.4% [n = 1633/4896]) and after (29.6% [n = 934/3155]) RV1 introduction. The relative abundance of rotavirus genotypes varied annually (Figure 1). The numbers of observed genotypes (richness) per year was higher after RV1 introduction compared to the prevaccine period (*P* = .034); however, no significant differences were observed in the overall genotype diversity index between the pre– and post–RV1 introduction periods (Table 1). The increase in combined G and P genotype richness post–vaccine introduction was observed in children aged 12–23 months compared to the prevaccine period (*P* = .010; Table 1). When VP4 and VP7 genotypes were analyzed separately, significant differences were observed in richness and diversity indices for P types; these differences were greatest in children aged >12 months during the postvaccine period compared to the prevaccine period (Table 2 and Supplementary Table 1).

**Table 1.** Rotavirus Genotype Diversity by Age in the Pre- and Postvaccine Periods

Age, mo	Observed (Richness)			Simpson Diversity Index			Shannon Diversity Index		
	Pre-Vac	Post-Vac	*P* Value	Pre-Vac	Post-Vac	*P* Value	Pre-Vac	Post-Vac	*P* Value
<6	4.71	3.71	.211	2.76	3.19	.2872	1.11	1.19	.799
6–11	5.43	6	.493	2.97	2.94	.9131	1.24	1.29	.856
12–23	4.37	7	.010	2.75	3.55	.149	1.14	1.46	.067
24–59	1.7	2.43	.254	1.6	2.13	.2707	0.42	0.7	.229
0–59	7.07	9.57	.034	3.13	3.42	.443	1.33	1.54	.110

The number of observed genotypes per year (richness) and differences in Shannon diversity index and Simpson diversity index were examined before (July 1997–October 2012) and after (November 2012–October 2019) vaccine introduction. Prevaccine genotypes (n = 1385) reported from previous studies [14, 16–20] and post–RV1 introduction genotypes (n = 693) in Table 2 were used. All samples with mixed and partially typed G or P genotypes were excluded.

Abbreviations: Post-Vac, postvaccination period; Pre-Vac, prevaccination period.

**Table 2.** Rotavirus P Genotype Diversity by Age in the Pre- and Postvaccine Periods

Age, mo	Observed (Richness)			Simpson Diversity Index			Shannon Diversity Index		
	Pre-Vac	Post-Vac	*P* Value	Pre-Vac	Post-Vac	*P* Value	Pre-Vac	Post-Vac	*P* Value
<6	2.5	2.71	.385	2.08	2.34	.247	0.76	0.91	.313
6–11	2.66	3.25	.063	2.06	2.38	.192	0.82	0.96	.093
12–23	2.62	3.6	.003	2.03	2.53	.023	0.74	1.04	.006
24–59	1.5	2.14	.065	1.38	1.89	.040	0.31	0.65	.031
0–59	2.38	2.89	.004	1.93	2.92	.002	0.68	0.89	.002

The number of observed single P genotypes per year (richness) and differences in Shannon diversity index and Simpson diversity index were examined before (July 1997–October 2012) and after (November 2012–October 2019) vaccine introduction. Prevaccine genotypes (n = 1420) reported from previous studies [14, 16–20] and post–RV1 introduction genotypes (n = 789) in Table 2 were used. All samples that had mixed P genotypes or were partially typed (assigned only G genotypes) were excluded.

Abbreviations: Post-Vac, postvaccination period; Pre-Vac, prevaccination period.

### Trends in the Distribution of Rotavirus Genotypes (July 1997–October 2019)

With the exception of G1 strains (associated with either P[4], P[6], or P[8]), that consistently circulated from 1997 to 2018, the distribution and relative proportion of other genotypes fluctuated over time (Figure 1). For instance, G3P[4], G3P[6], G3P[8], G4P[4], and G4P[6] were detected between 1997 and 1998, and then reemerged in 2017, whereas G4P[8] and G8P[6] prevailed from 1997 to 2007. Genotypes G8P[8], G9P[6], and G9P[8] circulated during 2001–2009 and then reemerged in 2018. G12 strains were detected for the first time in 2005, of which G12P[6] was the first to appear, followed by G12P[8] in 2007 and then by G12P[4] in 2012. Although G12 strains associated with P[9], P[11], and P[14] were detected sporadically between 2016 and 2019, G12 rotaviruses were highly prevalent between 2005 and 2015. Last, G2 rotaviruses (associated with either P[4], P[6], or P[8]) were first detected in 2008, were the predominant rotaviruses identified during the early postvaccine period, and were highly prevalent until 2018. By the end of 2017, strains that prevailed in the 1990s, such as G3, G8, and G9s, had reemerged (Figure 1). Genotypes G8P[4] and G8P[6] were frequently detected before RV1 introduction, while the detection rate of G1P[4], G2P[4], G2P[6], G2P[8], and G12P[8] was significantly higher in the postvaccine period (Table 3).

**Table 3.** Differentially Abundant Rotavirus Genotypes Between the Pre– and Post–RV1 Introduction Periods

Period	Rotavirus Genotypes													
	G1P[4] (%)	G1P[6] (%)	G1P[8] (%)	G2P[4] (%)	G2P[6] (%)	G3P[4] (%)	G3P[8] (%)	G8P[4] (%)	G8P[6] (%)	G8P[8] (%)	G9P[6] (%)	G9P[8] (%)	G12P[6] (%)	G12P[8] (%)
Pre-Vac	3 (0.20)	99 (7.41)	546 (38.38)	69 (4.03)	29 (1.64)	1 (0.09)	93 (4.50)	109 (9.33)	176 (10.29)	31 (3.61)	70 (9.25)	23 (1.75)	79 (5.54)	16 (1.49)
Post-Vac	14 (1.96)	30 (3.56)	184 (22.71)	184 (24.53)	75 (9.37)	47 (10.53)	22 (6.55)	3 (0.71)	0 (0)	5 (1.39)	5 (1.27)	5 (1.02)	61 (7.58)	31 (4.48)
*P* Value	.003	.076	.086	.006	.006	.178	.870	.038	.010	.457	.546	1.000	.408	.063

The indicator species analysis (IndVal) was performed to determine the genotypes that were differentially abundant by vaccination period before (July 1997–October 2012) and after (November 2012–October 2019) vaccine introduction. Wilcoxon signed-rank test was used. Only genotypes that were detected in >1.5% of the total characterized samples in either pre- or post-RV1 periods were included.

Abbreviations: Post-Vac, postvaccination period; Pre-Vac, prevaccination period.

**Figure 2.**
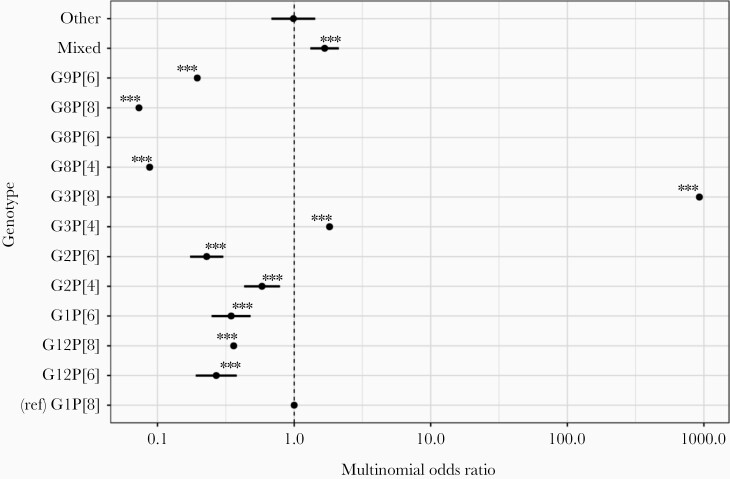
Multinomial odds ratios (MORs) for genotypes occurring before and after rotavirus vaccine introduction, November 1997–October 2019 (prevaccine period, n = 1422; postvaccine period, n = 896). Only full surveillance years were included in the multinomial regression analyses; therefore, rotavirus-positive genotyped samples from July to October 1997 (n = 84) and November 2009 to October 2011 (n = 7) were excluded from these analyses. Any single genotype that contributed ≤1.5% of samples in the whole study period was classified into a generic “other” category for “rarer” genotypes. *P* values for MORs: ***P < .001. G8P[6] was included in the model but not presented in Figure 2 because of extreme values related to there being no detections in the postvaccine era (adjusted MOR < 0.001).

After adjusting for surveillance year, age, and sex, the adjusted MOR (aMOR) of infection caused by G3P[8] (aMOR, 1038 [95% CI, 1001–1078]; *P* < .001) and G3P[4] (aMOR, 4.01 [95% CI, 4.01–4.02]; *P* < .001) was higher in the postvaccine period relative to wild-type G1P[8]. In contrast, cases due to G12P[6], G12P[8], G1P[6], G2P[4], and G2P[6] were lower in the postvaccine period relative to G1P[8], whereas cases due to mixed or other rarer genotypes were unchanged relative to G1P[8] (Figure 2).

### Genotype Distribution Post–RV1 Introduction (November 2012– October 2019)

Among the 934 rotavirus-positive stool samples detected following RV1 introduction, 24 distinct genotypes were identified, of which G1P[8], G2P[4], G2P[6], G12P[6], G3P[4], G12P[8], G1P[6], and G3P[8] were the most common (Table 4). Overall, G1, G2, and G12 rotaviruses were the predominant strains during the first 5 years postvaccination, but their prevalence declined with each successive year. From 2012 to 2017, the most common genotypes were G1P[8] and G2P[4], which alternated bi-ennially in predominance from 2012 to 2017 (Supplementary Figure 1). Genotype G3 strains associated with either P[4], P[6], or P[8] were first detected in the postvaccination period in November 2017; of all strains detected in 2018 and 2019, 36.3% and 27.2% comprised G3P[4] and G3P[8], respectively. By the end of 2019, G3 strains had replaced G1 and G2 strains as the predominant genotypes (Figure 1 and Table 4).

**Table 4.** Number of Rotavirus Specimens Collected During the Postvaccine Period

Genotype	Nov 2012–Oct 2013	Nov 2013–Oct 2014	Nov 2014–Oct 2015	Nov 2015–Oct 2016	Nov 2016–Oct 2017	Nov 2017–Oct 2018	Nov 2018–Oct 2019	Total (2011–2019)
	(n = 221/695)	(n = 128/495)	(n = 158/588)	(n = 125/397)	(n = 118/382)	(n = 103/360)	(n = 81/238)	(n = 81/238)
G1P[4]	0.5%	0.8%	2.5%	2.4%	3.4%	1.0%	…	1.34%
G1P[6]	1.8%	0.8%	15.8%	…	…	…	…	2.77%
G1P[8]	29.0%	12.5%	29.1%	10.4%	33.9%	4.9%	…	19.27%
G1P[14]	…	…	0.6%	…	…	…	…	0.09%
G2P[4]	18.6%	32.8%	3.8%	51.2%	21.2%	5.9%	…	20.70%
G2P[6]	10.4%	3.9%	1.3%	22.4%	11.9%	2.9%	…	8.83%
G2P[8]	0.5%	0.8%	…	1.6%	3.4%	1.0%	…	0.98%
G3P[4]	…	…	…	…	…	36.3%	12.3%	4.19%
G3P[6]	…	…	…	…	…	…	1.2%	0.09%
G3P[8]	…	…	…	…	…	…	27.2%	1.96%
G3P[10]	…	…	…	…	…	1.0%	…	0.09%
G4P[6]	…	…	0.6%	…	…	…	…	0.09%
G8P[4]	…	…	…	…	…	2.0%	1.2%	0.36%
G8P[8]	…	…	…	…	…	1.0%	4.9%	0.45%
G9P[4]	0.5%	…	…	…	…	…	1.2%	0.18%
G9P[6]	…	0.8%	…	…	…	1.0%	3.7%	0.45%
G9P[8]	…	…	…	…	1.7%	2.0%	1.2%	0.45%
G9P[9]	…	…	…	…	…	…	1.2%	0.09%
G12P[4]	…	…	0.6%	1.6%	…	1.0%	0.0%	0.45%
G12P[6]	8.1%	13.3%	15.2%	1.6%	…	…	0.0%	7.40%
G12P[8]	7.2%	…	1.3%	…	0.8%	8.8%	3.7%	3.39%
G12P[9]	…	…	…	…	…	…	1.2%	0.09%
G12P[11]	0.5%	…	…	…	…	…	…	0.09%
G12P[14]	…	…	3.2%	…	…	…	…	0.45%
Mixed^a^ and untypable	23.1%	34.4%	25.9%	8.8%	23.7%	31.4%	40.7%	25.78%

Specific genotypes detected in each year and proportions of rotavirus-positive stools, November 2012–October 2019.

^a^Results represent percentage of specific genotypes detected out of all genotypes characterized in rotavirus-positive stool samples collected (No.) in each November–October calendar year.

The median age of children infected with any genotype, with the exception of genotype G9P[8], was higher after RV1 introduction (Supplementary Table 2). The genotype distribution among different age groups was generally similar; however, G12 strains were rarely detected among children aged >23 months, and genotype G3P[8] was detected only in children aged 6–23 months. The proportion of mixed genotype infections increased in children aged <6 months in each consecutive year following RV1 introduction (Supplementary Figure 2).

## Discussion

A rich diversity of rotavirus strains was detected among children hospitalized with diarrhea in Blantyre during >22 years of surveillance. While we observed differences in the prevalence of individual rotavirus genotypes between pre– and post–RV1 vaccine periods, overall combined G and P genotype diversity in the 2 periods was similar. Genotypes G1P[8] and G2P[4] predominated just prior to RV1 introduction and thereafter declined in each successive year until 2018, after which they were not detected. In contrast, rotavirus strains that prevailed in the 1990s reemerged post-2018, notably G3, which became the predominant genotype by the end of 2019.

Unlike in most high-income countries, a wide rotavirus strain diversity circulates simultaneously in many LMICs [10], where atypical rotavirus strains such as G1P[6], G8P[4], G8P[6], G8P[8], and G9P[6] are frequently detected [7, 10, 21, 32]. In Malawi, up to 15 distinct rotavirus strains circulated within a single year (Figure 1). However, the impact of genotype diversity on vaccine performance remains unclear. Early studies suggested that strain diversity does not play a major role in determining vaccine efficacy [33], but the variability in rotavirus vaccine effectiveness against different rotavirus strains demonstrated by postvaccine introduction studies suggests that there may be differences in protection against rotavirus disease caused by homotypic compared with heterotypic strains. Thus, RV1 pooled vaccine effectiveness against homotypic strains is higher compared to partially heterotypic or fully heterotypic strains both in high- and low-income countries [34]. This was also demonstrated in Malawi, where RV1 was more effective against genotype G1 (vaccine effectiveness, 70.7%) compared to G2 (45.9%) and G12 (51.0%) strains [5].

Despite genotype-specific differences in vaccine effectiveness, it seems unlikely that the changes in rotavirus genotype distributions following vaccine introduction in Malawi could be attributed to selective vaccine pressure. For example, with each successive postvaccination year, G1 and G2 rotaviruses declined in frequency and they were not detected after 2018; if the frequency of circulating strains was vaccine driven, a gradual and constant decline in the detection of G1 compared to G2 and G12 rotaviruses would have been expected. It is noteworthy, however, that changes in genotype distributions following vaccination have been observed in other settings, in particular a predominance of G2 strains following introduction of RV1 in South America, the Caribbean, Australia, and some European counties [18–20, 35], which could be a result of heterogeneity in the vaccine-induced responses [36]. A lower observed vaccine effectiveness or a higher force of rotavirus infection [5, 37] could explain why a shift in combined G and P genotype diversity has not yet been observed in Malawi post–RV1 introduction, especially in children <12 months of age. Indeed, the cyclic pattern of circulating rotavirus strains in Malawi further suggests that the genotype changes were not driven by vaccine pressure but rather by a build-up and decline of homotypic immunity driven by natural rotavirus infection [38]. For example, genotype G2 strains emerged in 2008, 4 years before vaccine introduction, and then could not be detected 10 years later in 2018, similar to the 10-year cyclic circulation pattern observed for G2 rotaviruses in South Africa [39].

Regardless of the changes in genotype distributions, the overall prevalence of rotavirus has decreased substantially in all countries following rotavirus vaccine introduction. While in this study there was only a small decrease in the proportion of hospitalized diarrheal cases that tested positive for rotavirus following vaccine introduction among children aged <5 years, the greatest reduction in the prevalence of rotavirus in this population has been reported in those aged <12 months [40]. Furthermore, while we did not observe differences in the age distribution of children infected with different genotypes, the mean age of rotavirus cases increased during the vaccine period regardless of the infecting strain, providing additional evidence of vaccine impact [5]. Although there was no significant difference in the overall combined rotavirus G and P genotype diversity between the pre- and postvaccine periods, the diversity indices for P genotypes increased during the postvaccine period in children aged >12 months, similar to what was observed in children in England aged >6 months for combined G and P types [35]. Several possibilities may explain this observation. In Malawian children, a higher vaccine effectiveness (70.6%) is observed during the first year of life compared to the second year of life (31.7%), and RV1 homotypic protection against G1P[8] genotypes is higher compared to non-G1P[8] heterotypic protection [5]. This, coupled with a high force of rotavirus infection in Malawi [37], likely exposes children to multiple genotypes as they grow older. Thus, with the lower vaccine-induced heterotypic protection, non-G1P[8] rotavirus genotypes would have increased fitness among vaccinated children, thereby permitting them to persist in children aged >12 months until most have been exposed to multiple different strains. Further studies are warranted to investigate why, following vaccine introduction, diversity increased only in VP4 genotypes and not in VP7 genotypes. Nevertheless, these findings support the role of VP4 in mediating vaccine-induced protection and supports the use of recombinant VP5* (the stalk region of VP4) as useful targets for the development of improved third-generation rotavirus vaccines [41, 42].

Our study has some limitations. The diversity of genotypes that circulated prior to vaccine introduction could have been underestimated compared to the postvaccine years, as the RT-PCR assay used during the post–RV1 introduction period included additional genotype-specific primers. We did not determine the frequency of the occurrence of nonspecific PCR product or PCR artifacts resulting from primer cross-binding or mismatches, which could have potentially resulted in overestimation of the proportion of mixed infections during the postvaccine era due to additional primers included in the genotyping cocktail [43]. It is likely, though, that the frequency of PCR artifacts was low, as all primers that were utilized are well validated and are used worldwide to genotype rotaviruses [27]. Nevertheless, our study provides a useful temporal observation of rotavirus strain variation and is one of the longest reported descriptions of rotavirus strains globally and, to our knowledge, the longest from a single site in Africa.

In conclusion, our comprehensive rotavirus strain surveillance dataset spanning more than 2 decades demonstrates that genotype diversity did not significantly change following introduction of rotavirus vaccine. The overall variation in the prevalent rotavirus genotypes could not be attributed to vaccine introduction and most likely represents natural genotype oscillation. These data support continued use of rotavirus vaccination regardless of the circulating rotavirus genotypes. Nevertheless, continued strain surveillance is warranted to accompany ongoing vaccine impact evaluation and to monitor viral evolution.

## Supplementary Data

Supplementary materials are available at *The Journal of Infectious Diseases* online. Consisting of data provided by the authors to benefit the reader, the posted materials are not copyedited and are the sole responsibility of the authors, so questions or comments should be addressed to the corresponding author.

jiaa616_suppl_Supplementary_Table_1

jiaa616_suppl_Supplementary_Table_2

jiaa616_suppl_Supplementary_Figure_1

jiaa616_suppl_Supplementary_Figure_2

## Notes

Presented in part: 12th African Rotavirus Symposium, Johannesburg, South Africa, 30 July–1 August 2019; and 23rd College of Medicine Research Dissemination Conference, University of Malawi, Blantyre, Malawi, 7–8 November 2019.

***Author contributions.*** K. C. J., M. I.-G., and N. A. C. conceptualized the project. N. A. C. and K. C. J. facilitated the sample resources. Data curation and validation were performed by R. W., J. J. M., D. H., A. W. K., C. M., and K. C. J. Laboratory experiments were performed by C. M., J. J. M., E. C., O. C., C. M.-B., P. M., I. S., and K. C. J. Formal analyses were performed by C. M., J. J. M., A. W. K., C. C., D. H., and K. C. J. Writing of the original draft preparation was performed by K. C. J., M. I.-G., and N. A. C. Review of the drafts was performed by all coauthors. Supervision, investigations, and project administration were performed by K. C. J. and N. A. C. Funding acquisition was performed by K. C. J., N. A. C., U. D. P., M. M. N., and V. E. P. All authors read and approved the final manuscript.

***Acknowledgments.*** We acknowledge the support of the laboratory staff at the Malawi-Liverpool-Wellcome Trust Clinical Research Programme, the clinical team, and the study participants. We thank Dr Aisleen Bennett, Dr Louisa Pollock, and Dr Naor Bar-Zeev for their role in collection of clinical data and fecal samples; and Billy Khembo, Sheilla Kafuwa, and Yohane Chisale for their contribution in genotyping some of the strains included in this analysis.

***Disclaimer.*** The funders had no role in the study design, data collection and interpretation, or the decision to submit the work for publication. The authors received no financial support or other form of compensation related to the development of the manuscript. The findings and conclusions in this report are those of the authors and do not necessarily represent the official position of the US Centers for Disease Control and Prevention (CDC), the National Health Service, the National Institute for Health Research (NIHR), the United Kingdom Department of Health, or Public Health England (PHE).

***Financial support.*** This work was supported by research grants from the Wellcome Trust (program grant number 091909/Z/10/Z and International Training Fellowship number 201945/Z/16/Z); the Bill & Melinda Gates Foundation (grant number OPP1180423); CDC funds through World Health Organization (WHO) (grants 2018/815189-0 and 2020/1004857-0); and NIH/NIAID (grant number: R01AI112970). M. I.-G., D. H., and N. A. C. are affiliated with the NIHR Health Protection Research Unit in Gastrointestinal Infections at the University of Liverpool in partnership with PHE, in collaboration with the University of Warwick. K. C. J. is a Wellcome International Training Fellow. D. H. is funded by an NIHR Post‐Doctoral Fellowship (PDF‐2018‐11‐ST2‐006).

***Potential conflicts of interest.*** K. C. J. and N. A. C. have received investigator-initiated research grant support from the GSK group of companies. M. I.-G. has received investigator-initiated research grant support from the GSK group of companies, Sanofi Pasteur, Merck Sharpe & Dohme, and Merck. N. A. C. has received research grant support and honoraria for participation in rotavirus vaccine data safety monitoring committee meetings from the GSK group of companies. V. E. P. is a member of the WHO Immunization and Vaccine-Related Implementation Research Advisory Committee and has received reimbursement from Merck and Pfizer for travel expenses to attend scientific input engagements unrelated to the subject of this manuscript. All other authors report no potential conflicts.
