# Supplementary material for: Rotavirus Genotypes in Hospitalized Children With Acute Gastroenteritis Before and After Rotavirus Vaccine Introduction in Blantyre, Malawi, 1997–2019
Source: J Infect Dis. 2020 Oct 9;225(12):2127–36. doi: 10.1093/infdis/jiaa616 (PMC9200156; doi:10.1093/infdis/jiaa616)
Supplement: jiaa616_suppl_Supplementary_Table_1 [file jiaa616_suppl_supplementary_table_1.docx]

**Supplementary Table S1. Rotavirus G genotype diversity by age in the pre- and post-vaccine periods.** The number of observed genotypes per year (richness), differences in Shannon diversity (H) index and Simpson diversity (D) index were examined before (July 1997 – October 2012) and after (November 2012 – October 2019) vaccine introduction. Pre-vaccine genotypes (n=1,404) reported from previous studies [14, 16-20] and post-RV1 introduction genotypes (n=796) in Table 4 were used. All samples that had mixed G genotypes or were partially typed (assigned P genotypes only) were excluded.

|  | **Observed (Richness)** | | | **Simpson Diversity (D’)** | | | **Shannon Diversity (H’)** | | |
| --- | --- | --- | --- | --- | --- | --- | --- | --- | --- |
| **Age (months)** | ***Pre-Vac*** | ***Post-Vac*** | ***P-value*** | ***Pre-Vac*** | ***Post-Vac*** | ***P-value*** | ***Pre-Vac*** | ***Post-Vac*** | ***P-value*** |
| **<6** | 2.79 | 2.86 | 0.635 | 1.91 | 2.26 | 0.296 | 0.69 | 0.87 | 0.332 |
| **6 – 11** | 3.21 | 3.71 | 0.293 | 2.1 | 1.99 | 0.799 | 0.82 | 0.84 | 0.856 |
| **12 – 23** | 2.86 | 4.14 | 0.041 | 1.94 | 2.51 | 0.225 | 0.74 | 1 | 0.287 |
| **24 – 59** | 1.4 | 1.86 | 0.171 | 1.34 | 1.66 | 0.213 | 0.26 | 0.499 | 0.213 |
| **0 – 59** | 2.65 | 3.14 | 0.090 | 1.86 | 2.1 | 0.125 | 0.66 | 0.8 | 0.138 |
