# Supplementary material for: Rotavirus Genotypes in Hospitalized Children With Acute Gastroenteritis Before and After Rotavirus Vaccine Introduction in Blantyre, Malawi, 1997–2019
Source: J Infect Dis. 2020 Oct 9;225(12):2127–36. doi: 10.1093/infdis/jiaa616 (PMC9200156; doi:10.1093/infdis/jiaa616)
Supplement: jiaa616_suppl_Supplementary_Table_2 [file jiaa616_suppl_supplementary_table_2.docx]

**Supplementary Table S2: Median age of children infected with specified genotype before (July 1997 – October 2012) and after (November 2012 – October 2019) vaccine introduction.** Age was missing for 20 pre-vaccine and 10 post-vaccine study participants that tested positive for rotavirus. IQR represents interquartile range. NA represents indeterminant p-values due to insufficient number of samples during either before or after vaccine introduction period. All RV+ represents all stool samples that were rotavirus-positive on EIA.

|  | **Pre-Vaccine Period** | | | **Post-Vaccine Period** | | | |
| --- | --- | --- | --- | --- | --- | --- | --- |
| **Genotype** | ***n^a^*** | **Median** | **IQR** | ***n^b^*** | **Median** | **IQR** | ***p*-value** |
| **G1P[6]** | 98 | 8.2 | 6.7 (5.6 – 12) | 29 | 12 | 7 (8 – 15) | 0.004 |
| **G1P[8]** | 543 | 7.8 | 6.3 (5.3 – 11.7) | 183 | 10 | 6 (8 – 14) | <0 .001 |
| **G2P[4]** | 64 | 6.5 | 4.5 (4.5 – 11) | 180 | 10 | 6 (8 – 14) | 0.002 |
| **G2P[6]** | 28 | 6 | 6.5 (3 – 947) | 76 | 9 | 6.3 (7 – 13.3) | 0.001 |
| **G3P[4]** | 1 | - | - | 47 | 11 | 5 (8 – 13) | NA |
| **G3P[6]** | 12 | 5.8 | 5.8 (4 – 9.8) | 1 | - | - | NA |
| **G3P[8]** | 93 | 7.9 | 5.6 (6 -11.6) | 21 | 9 | 3 (7 – 10) | 0.171 |
| **All G3s** | 183 | 8.3 | 6.1 (5.7 – 11.7) | 71 | 10 | 5 (8 – 13) | 0.001 |
| **G8P[4]** | 109 | 8.6 | 6.3 (5.6 – 12) | 3 | 16 | 17.5 (8 – 25.5) | <0.001 |
| **G8P[8]** | 31 | 9.1 | 6 (5.7 – 11.8) | 5 | 14 | 2.5 (12 – 14.5) | 0.001 |
| **G9P[6]** | 70 | 8 | 6.5 (5.4 -11.9) | 5 | 15 | 11.5 (7.5 – 19) | 0.068 |
| **G9P[8]** | 23 | 10.7 | 4.4 (8.2 – 12.6) | 5 | 15 | 17.5 (28 – 10.5) | 0.011 |
| **G12P[6]** | 79 | 5.6 | 6 (3.4 – 9.4) | 60 | 9 | 6.3 (6 – 12.3) | 0.013 |
| **G12P[8]** | 16 | 6.7 | 4 (4.8 – 8.8) | 30 | 9 | 6.3 (6.8 – 13) | 0.038 |
| **All RV+** | 1623 | 7 | 6 (5 -11) | 924 | 10 | 6.8 (7.3 – 14) | <0.001 |

***^a^***273 pre-vaccine samples contained either mixed genotypes, were partially typed or not successfully genotyped hence excluded from the analysis.

***^b^***208 post-vaccine samples contained either mixed genotypes, were partially typed or not successfully genotyped hence excluded from the analysis.
