# Supplementary material for: Rotavirus Genotypes in Hospitalized Children With Acute Gastroenteritis Before and After Rotavirus Vaccine Introduction in Blantyre, Malawi, 1997–2019
Source: J Infect Dis. 2020 Oct 9;225(12):2127–36. doi: 10.1093/infdis/jiaa616 (PMC9200156; doi:10.1093/infdis/jiaa616)
Supplement: jiaa616_suppl_Supplementary_Figure_1 [file jiaa616_suppl_supplementary_figure_1.pdf]

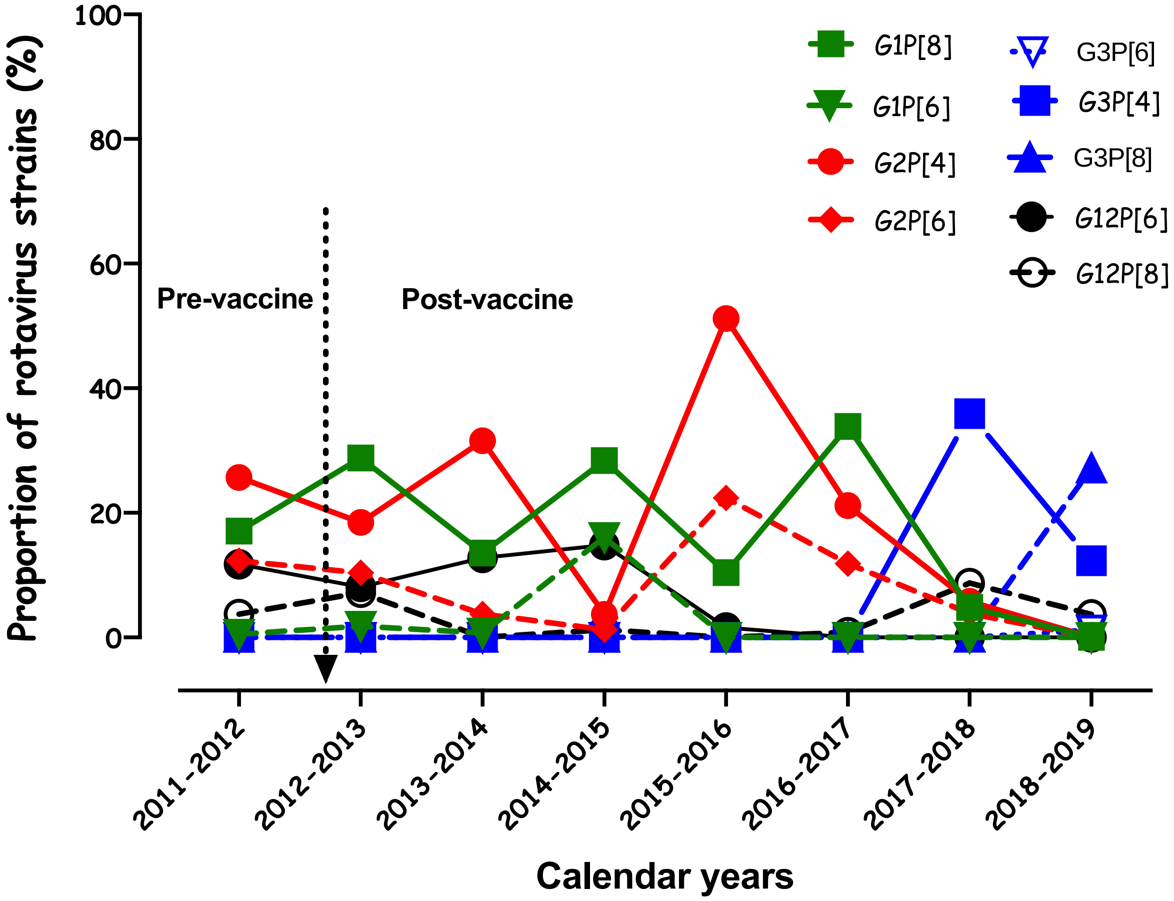

Supplementary Figure S1. Trends of rotavirus genotypes from December 2011 – November 2019. Data from 2012 are presented in November – December calendar years. The arrow Rotarix® vaccine introduction. Green represents genotype G1, red represents G2, blue represents G3 and black represents G12.
