# Supplementary material for: Rotavirus Genotypes in Hospitalized Children With Acute Gastroenteritis Before and After Rotavirus Vaccine Introduction in Blantyre, Malawi, 1997–2019
Source: J Infect Dis. 2020 Oct 9;225(12):2127–36. doi: 10.1093/infdis/jiaa616 (PMC9200156; doi:10.1093/infdis/jiaa616)
Supplement: jiaa616_suppl_Supplementary_Figure_2 [file jiaa616_suppl_supplementary_figure_2.pdf]

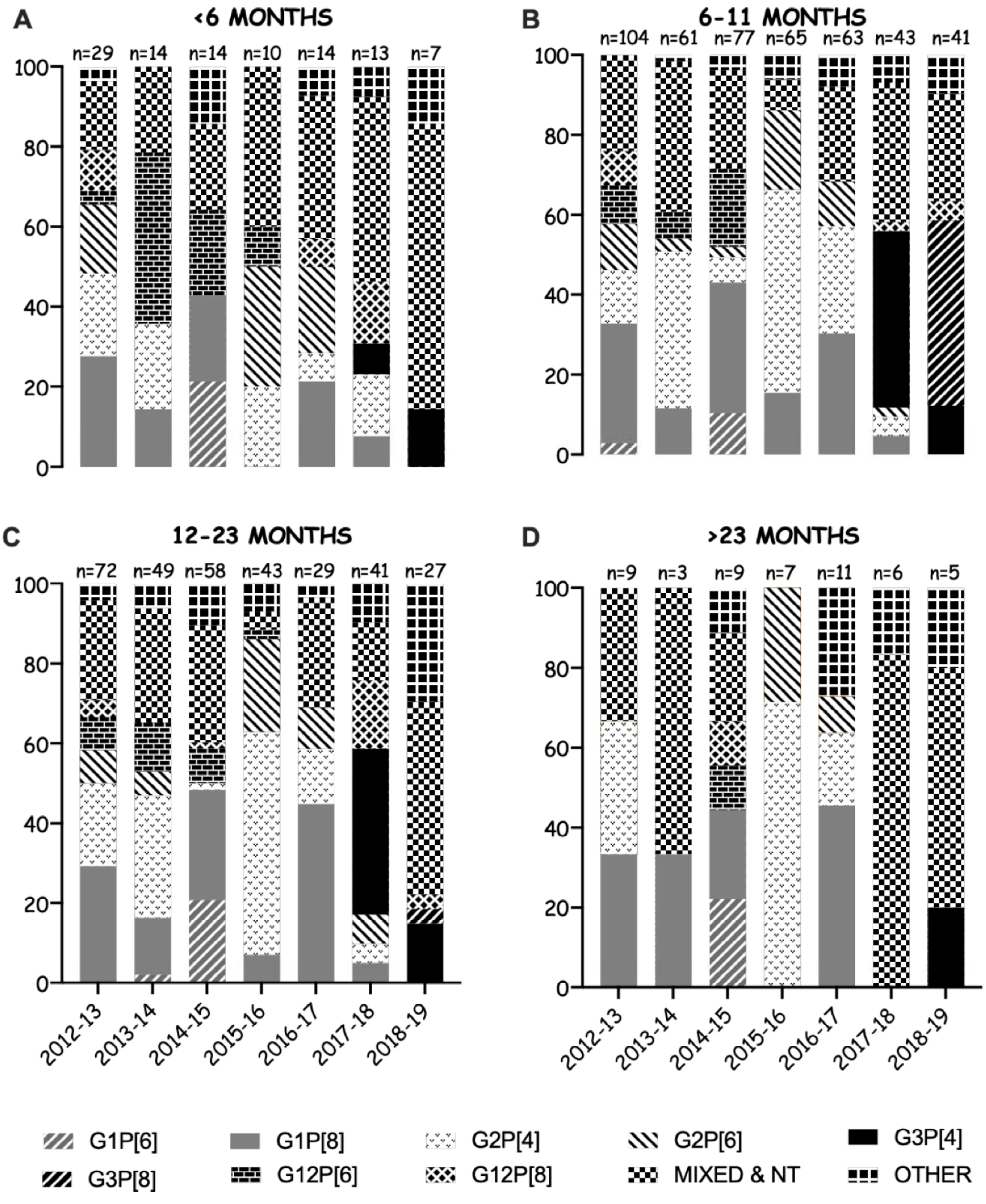

Supplementary Figure S2. Proportion of rotavirus genotypes detected in rotavirus-positive stool samples collected from children in different age groups during the post-vaccine period (Nov 2012 – Oct 2019). (A) Children < 6 months old. (B) Children 6 – 11 months old. (C) Children 12 – 23 months old. (B) Children > 23 months old. Others represents genotypes detected in $\leq$ 1.5% of the total cases and Mixed & NT represents samples that had either a mixture of G and P types or were not typeable; see Table 2.
